# Comparison of physiological demands in Warmblood show jumping horses over a standardized 1.10 m jumping course versus a standardized exercise test on a track

**DOI:** 10.1186/s12917-020-02400-9

**Published:** 2020-06-08

**Authors:** Renaud Léguillette, Stephanie L. Bond, Kelda Lawlor, Tineke de Haan, Lauren M. Weber

**Affiliations:** Faculty of Veterinary Medicine, Department of Veterinary Clinical and Diagnostic Services, University of Calgary, Calgary, Alberta T2N 4N1 Canada

**Keywords:** V170, V200, VLa4, Exercise physiology

## Abstract

**Background:**

A greater understanding of exercise physiology and biochemistry is required for the sport horse disciplines, including show jumping. Conditioning of horses for show jumping is empirical because they are primarily trained on flat ground, however the equivalent workload between jumping and flat work is currently unknown. The objectives of the study were therefore to compare the physiological demands of Warmblood show jumpers over a standardized 1.10 m course vs a 600 m standardized incremental exercise test on flat ground, and to report reference field test values for competitive show jumping horses. In this prospective field study, 21 healthy, actively competing Warmblood show jumping horses were assessed to determine physiological variables after a standardized jumping course at 6.4 m/s (average speed) and track standardized incremental exercise test at 5 m/s, 8 m/s and 11 m/s. Heart rate, velocity, blood lactate, blood pH, pCO2, bicarbonate, PCV and TP concentrations were recorded. V200, V170 and VLa4 were calculated. Parametric statistics were performed on analysis of all 21 horses’ variables.

**Results:**

Contrary to exercise at 5 m/s and 11 m/s, cantering at 8 m/s did not induce any significant difference in blood lactate, mean heart rate or mean venous blood pH compared to after completion of the jumping course.

**Conclusions:**

Jumping a 1.10 m course demands a statistically similar workload to cantering around a flat track at 8 m/s. This study will help to test fitness and design conditioning programs for Warmblood show jumping horses.

## Background

Physical conditioning of the equine athlete is a significant issue that has implications for both performance and welfare. A lack or an excess of physical conditioning predisposes racehorses to injury [1] and the same might be true for show jumping horses. Variables commonly used to evaluate equine fitness under field conditions include heart rate (HR), packed cell volume (PCV), total protein concentration (TP), acid-base balance (pH, pCO2, HCO3- concentration), electrolyte concentrations (Na^+^, K^+^, Ca^2+^), glucose concentration, blood (or plasma) lactate concentration and velocity [2–6]. The use of performance variables including heart rate and blood lactate have been utilized in standardized exercise tests to evaluate aerobic and anaerobic capacity in the field [7, 8] but little data is available to comparatively assess the fitness of show jumping horses. Show jumpers experience physiological changes as a result of jumping, jumping in a competition, and jumping with a change in fence height [5, 6, 9]. Warmblood show jumpers therefore utilize a specific balance of aerobic and anaerobic metabolic processes [6, 10]. Show jumpers are primarily conditioned using flat work exercise to decrease the stress imposed to the musculoskeletal system. However, as the equivalent workload between jumping and flat work is currently unknown, conditioning of these horses is empirical. It is therefore necessary to understand the equivalent exercise intensity between show jumping and flat work to condition jumpers properly.

The physiological demands of several equestrian disciplines including horse racing, endurance racing and three-day eventing have previously been investigated [11–14]. A greater understanding of exercise physiology and biochemistry is required for the sport horse disciplines, including show jumping. Although a few studies have been performed on show jumping horses, the difference in metabolic demands between flat and jumping work is unknown and needs to be addressed to assist trainers optimize fitness programs. Assessment of Warmbloods during a field exercise test can be difficult, as quite often the rider and trainer, as well as the horse, is unwilling to gallop at higher speeds. Whilst track tests suitable for Warmbloods and other horses competing in non-racing disciplines have been described [15–17], the use of V170, rather than V200 has been proposed for use in endurance and other saddle horses, due to closer representation of these discipline’s physiologic demands [18]. Such data is necessary to better prescribe workload and to better monitor the fitness of show jumping horses.

Our hypothesis was that a greater galloping speed would be required on a flat racetrack than on a 1 m10 jumping course to obtain a similar physiological workload. The aims of the present study were therefore to compare the physiological demands of Warmblood show jumpers over a standardized 1.10 m course vs a 600 m standardized incremental exercise test, and to report reference field test values for exercise physiology variables (V200, V170 and VLa4) in competitive show jumping Warmblood horses.

## Results

### Descriptive analysis

The average velocities, heart rates, blood lactate concentrations, blood pH concentrations, pCO_2_ concentrations, bicarbonate concentrations, PCV and TP concentrations for both the standardized jumping course and the standardized track fitness test are reported in Table 1.

**Table 1:** Summary of the average velocities, heart rates, blood lactate concentrations, pH concentrations, pCO2 concentrations, bicarbonate concentrations, PCV and TP concentrations for the jumping phase, after a 60-min recovery, and the 600 m fitness test. All values are shown as mean ± S.D. § indicates significantly different from jumping

	After Warmup 1	After Jumping	60-min recovery	After Warmup 2	5 m/s flat canter	8 m/s flat canter	11 m/s flat gallop
Velocity (m/s)	See methods	6.42 ± 0.35^a^	NA	See methods	5.31 ± 0.38	7.79 ± 0.57	10.68 ± 0.70
Heart Rate (beats/min)	NA	175.4 ± 13.4	NA	NA	139.3 ± 12.9 §	161.5 ± 36.6	196.8 ± 9.6 §
Blood Lactate (mmol/l)	0.2 ± 0.2 §	2.7 ± 1.3	0.8 ± 0.3 §	0.1 ± 0.04 §	0.6 ± 0.2 §	2.4 ± 0.7	7.4 ± 2.1§
pH	NA	7.48 ± 0.03	7.47 ± 0.02	NA	7.54 ± 0.04	7.51 ± 0.04	7.41 ± 0.05
pCO_2_ (mmol/l)	NA	38.7 ± 3.6	40.2 ± 2.7	NA	35.5 ± 3.5	36.3 ± 3.6	41.5 ± 5.8
Bicarbonate (mmol/l)	NA	28.4 ± 2.1	29.1 ± 1.8	NA	29.9 ± 1.7	28.4 ± 1.4	25.9 ± 2.6
PCV (l/l)	NA	44.4 ± 7.0	35.8 ± 2.8	NA	46.9 ± 2.3	49.9 ± 2.4	53.6 ± 2.2
TP (g/l)	NA	76.1 ± 6.1	71.4 ± 5.9	NA	75.5 ± 4.2	75.7 ± 4.5	78.6 ± 4.3

^a^Average velocity (m/s) during jumping course

The average time required to complete the standardized jumping course without refusals was 59.45 ± 3.43 s overall, with an average speed of 6.42 ± 0.35 m/s.

**Fig. 1:**
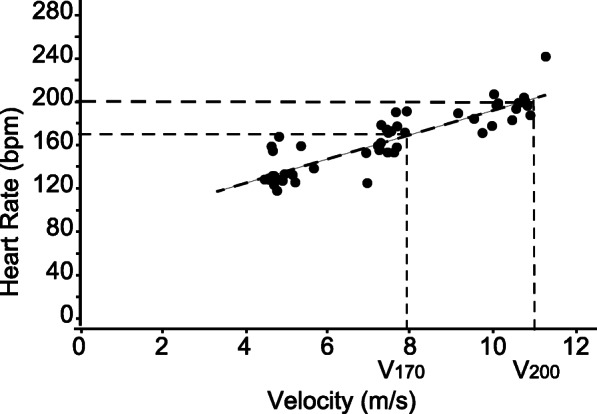
Incremental standardized track test in 21 show jumping Warmblood horses using a heart rate monitoring and GPS cell phone equipment: The velocity (m/s) calculated from the standardized exercise test on the track (target speeds of 5, 8 and 11 m/s) at which the heart rate will be: 170 beats/min (V170: 7.9 ± 1.1 m/s) and 200 beats/min (V200: 11.1 ± 0.9 m/s)

Mean values for the heart rate obtained during the track standardized exercise test were plotted as a function of velocity, enabling the calculation of V200 and V170 (Fig. 1). The slopes calculated for the linear regression lines of HR to speed for each horse were then averaged; resulting in a mean V200 of 11.1 ± 0.9 m/s and V170 of 7.9 ± 1.1 m/s (Fig. 1).

**Fig. 2:**
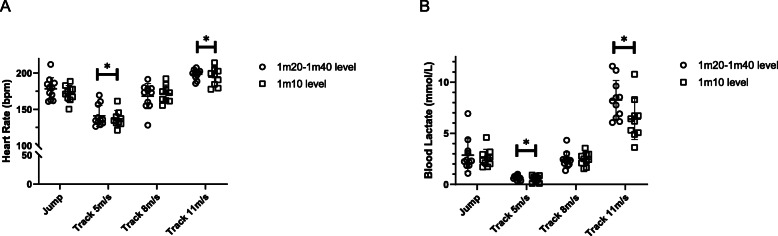
Heart rate (**a**) and blood lactate (**b**) values in 21 show jumping Warmblood horses immediately after a standardized jumping test and an incremental standardized track test (target speeds of 5, 8 and 11 m/s). The squares show horses competing in the lower (1 m10) category and circles show horses competing at a high level (1 m20- 1 m40). Mean and SD are represented by the lines in the graph. * indicates significantly different from the jumping test

Similarly, the mean values for blood lactate obtained during the track standardized exercise test (Table 1, Fig. 2) were plotted as a function of velocity and a mean VLa4 of 9.5 ± 0.5 m/s was obtained.

The mean blood lactate concentration collected immediately after jumping was 2.7 ± 1.3 mmol/l (Fig. 2). It decreased significantly 60 min after jumping to 0.8 ± 0.3 mmol/l (*p* < 0.0001).

The mean PCV and TP at the conclusion of the jumping course were 44.4 ± 7.0 l/l and 76.1 ± 6.1 g/l respectively. The PCV and TP 60 min after completion of jumping were 35.8 ± 2.8 l/l and 71.4 ± 5.9 g/l respectively. The mean PCV, but not the TP, increased significantly (p < 0.0001) from the 5 m/s through to the 11 m/s velocity (Table 1). The mean PCV increased by 49.5%, and the mean TP increased by 10.0% between the 60-min recovery time after jumping and the fastest part of the standardized exercise test at a gallop of 11 m/s on the track.

### Comparison between the 1.10 m competition and the 1.20–1.40 m competition groups

To compare the physiological response of higher versus lower level show jumping horses, 21 horses were enrolled, 10 of which competed at the 1.10 m level and 11 competed between 1.20 m and 1.40 m. There was no significant difference in V200, V170, VLa4. Furthermore, there was no statistical difference in the slope of the linear regression line of HR to speed, rectal and ambient temperatures during the standardized exercise test between the two groups. The analysis showed that the standardized exercise tests had a significantly different effect on the horses from the lower vs the higher jumping level category on blood lactate, pH, and pCO_2_, but not on bicarbonates, PCV and total protein.

There was no significant difference in mean blood lactate at 5 m/s or 8 m/s between the 1.10 m and 1.20–1.40 m competition groups, however at 11 m/s, the blood lactate and pCO_2_ of the 1.20–1.40 m competition horses (8.27 ± 1.89 mmol/l and 44.25 ± 1.5 mmol/l, respectively) was significantly greater and the blood pH (7.38 ± 0.09) was significantly lower than the 1.10 m competition horses (6.40 ± 2.0 mmol/l, 38.5 ± 1.12 mmol/l and 7.43 ± 0.04, respectively) (*p* = 0.003 to 0.007).

### Comparison between the standardized jumping course and the track standardized exercise test

There was significant variability in blood lactate, pH, pCO_2_, bicarbonates, between horses.

There was no statistically significant interaction between the exercise tests and the horses’ competition groups and only the exercise tests had an effect on the mean heart rate. Multiple comparisons of the mean heart rate showed that measurements obtained during the jumping course (175.4 ± 13.4 beats/min) were significantly higher than during the 5 m/s canter (139.3 ± 12.9 beats/min; *p* < 0.0001), and significantly lower than during the 11 m/s gallop (196.8 ± 9.6 beats/min; *p* < 0.0001). There was no statistical difference in mean heart rate during the 8 m/s canter than during completion of the jumping course (Table 1, Fig. 2).

The mean blood lactate measurement collected after completion of the jumping course (2.7 ± 1.3 mmol/l) was significantly higher than after the 5 m/s canter (0.6 ± 0.2 mmol/l; *p* < 0.0001), and significantly lower than after the 11 m/s gallop (7.4 ± 2.1; *p* < 0.001). There was no statistical difference in blood lactate after the 8 m/s canter (2.4 ± 0.7 mmol/l) and after completion of the jumping course (Table 1, Fig. 2).

The mean venous blood pH after completion of the jumping course was 7.48 ± 0.03, which was not significantly different from the pH measured after the 8 m/s exercise (7.51 ± 0.04) but was different (p < 0.0001) from the 5 m/s and 11 m/s exercise tests (7.54 ± 0.04 and 7.41 ± 0.05, respectively).

The mean venous blood pCO_2_ after completion of the jumping course (38.7 ± 3.6 mmol/l) was only different (*p* = 0.008) from the venous blood pCO_2_ measured during after the 5 m/s exercise test (35.5 ± 3.5).

The mean venous blood bicarbonate concentration measurement obtained after completion of the jumping course (28.4 ± 2.1 mmol/l) was significantly different (*p* = 0.009 and *p* = 0.0002, respectively) from the concentrations measured after the 5 m/s and 11 m/s gallop (29.9 ± 1.7 and 25.9 ± 2.6 mmol/l, respectively).

The mean PCV obtained after completion of the jumping course (44.4 ± 7.0 l/l) was significantly lower (*p* < 0.0001) than the PCV obtained after completion of the 8 m/s canter and 11 m/s gallop (8 m/s: 49.9 ± 2.4 l/l, 11 m/s: 53.6 ± 2.2 l/l) but was not different from the PCV obtained after the 5 m/s canter (46.9 ± 2.3 l/l).

There was no statistical difference in the mean TP obtained immediately after completion of any of the exercises (jumping, 5 m/s, 8 m/s, and 11 m/s track tests). However, the mean TP at the 60 min recovery time point after jumping was significantly lower than immediately post jumping and after the 11 m/s track test (*p* = 0.0011).

## Discussion

This study investigated the physiological demands of Warmblood show jumpers using a standardized 600 m fitness test at three velocities and a standardized course over fences while measuring the horses’ heart rate and specific blood chemistry. Furthermore, we wanted to determine if there was a difference in the fitness levels between horses who had competed at 1.10 m and 1.20 m–1.40 m. The comparison between the two types of standardized exercise on a relatively high number of horses allowed for the description of exercise physiology variables (V200, V170, and VLa4) and for comparison of the metabolic response of Warmblood showjumpers on the track at set speeds versus their jumping effort on a 1.10 m course. This data is important as it provides guidance on conditioning and training protocols for show jumping horses in instances where training occurs primarily on the flat, where the speed necessary to reproduce a similar cardiovascular effort to show jumping competitions was previously unknown. In addition, the present study provides values for V200, V170, and VLa4 in competitive show jumping Warmbloods performing a standardized field test that can be utilized in the future for assessing and comparing the fitness of showjumpers in field conditions. The two main physiological variables usually used to estimate exercise intensity in field conditions are the heart rate and blood lactate accumulation (see discussion below); we found that only the track velocity of 8 m/s canter showed no significant difference in both variables when compared to the completion of the jumping course. Therefore, of the three tested flat track speeds, jumping a 1.10 m course demanded a workload in the sampled horses that was not different to cantering at 8 m/s. There was no statistical difference in V200/V170 between the 1.10 m and 1.20–1.40 m competition horses, and there was no effect of the horses’ competition level on the response to exercise of the HR or any of the blood parameters, indicating a similar level of aerobic conditioning between the two groups. The course design and recommended speed were provided by a certified course designer to be representative of a typical 1.10 m show jumping competition.

The 21 horses enrolled in the present study were all fit, experienced Warmblood performance horses of comparable age and jumping level (10 horses were competing at 1.10 m–1.20 m; 11 horses were competing at 1.20–1.40 m) that were all actively participating in the same competition circuit. The specifics of their training varied depending on the trainers’ preference, but all horses were performing as expected in the competitions.

Reference physiological values have been reported before in 18 amateur jumping and/or dressage (distribution between the 2 groups not described) Warmbloods using a high-speed treadmill protocol [19]. The differences in metabolic response between treadmill and track exercise make it difficult to compare both studies’ data sets, but the numbers obtained in our field study showed higher velocity values for all variables, probably indicating (as expected) that the performance Warmblood show jumpers enrolled in the present study were fitter than the amateur horses enrolled in the treadmill study [19]

A limitation of the present study is the inherent variability associated with field studies (weather, ambient temperature, track surface). Conversely, while performing a study on a high-speed treadmill allows for excellent repeatability, standardization of conditions and precision in the control of speed [19], it imposes a different workload and biomechanics of locomotion and does not account for the effect of a rider, compared to field testing [20–23]. Therefore, variability due to weather conditions were minimized by performing the study during the summer time. The variability due to rider was minimized by using the same rider for the jumping course as well as for the track test (except in 2/ 21 cases). In addition, the track was not used in heavy conditions and was groomed each morning before the tests so that variability was decreased. The effect warmup exercise could have on the data collection was considered and was therefore standardized for both the jumping and track exercise tests.

The mean heart rate measured during the 1.10 m jumping phase is slightly lower than the previously reported heart rate over 0.8–1.0 m high fences despite using a slightly greater speed (5.2 ± 0.2 m/s versus 6.42 ± 0.35 m/s in the present study) [5]. However, there were many differences between the two studies, primarily the use of riding-school horses versus performance show jumpers (present study). Although riding-school horses are exercised frequently, the workload tends to be low [24, 25]. The average heart rate over fences was in agreement with the mean heart rate range previously reported by Art et al. during an official 1.50 m championship competition [2]. The mean heart rate recorded during the jumping phase was not different from the mean heart rate during the 8 m/s canter. However, the average velocity over the jumping course was 6.42 ± 0.35 m/s, which is substantially slower than the velocity required to obtain the same heart rate on the track. Our finding, that jumping exercise imposes a greater workload than flat work, concurs with a previous report [5]; however the study design used presently also allowed for the evaluation of differences in exercise intensity. Indeed, the results suggest that to condition show jumpers when preparing them for a 1.10 m competition, flat work should be done at approximately 8 m/s to simulate the same intensity of workload without adding the stress of jumping to the musculoskeletal system [26]. It should be noted that reaching 8 m/s may still be considered as an unusually high speed in some show jumping horses and may be difficult to implement as a routine in training programs. The study highlights the fact that riders and trainers should be aware of the importance of increasing the workload during conditioning of show jumpers, for example by increasing their usual cantering speed on flat terrain or by using some slope work.

The results from several studies in eventing horses suggest that V200 might be an indicator of maximal performance and that VLa4 might be a better indicator of fitness; VLa4 progresses as horses compete at higher levels, however V200 is consistently reported to be between 10.8 and 11.8 m/s for horses at all eventing levels [15, 27, 28]. Therefore, heart rate data might not be the best indicator of fitness or workload for show jumping horses. However, when comparing blood lactate levels, there was no difference in blood lactate after the 8 m/s canter than after completion of the jumping course; the values were almost identical. This is concurrent with a previous report [5] which indicates that cantering the same distance without fences results in a lower blood lactate level than cantering the same distance with fences. The reported difference in blood lactate could be due to an increased workload; circulation to working muscles is altered due to an increase in oxygen demand during take-off and landing [5]. Indeed, at 11 m/s the blood lactate of the 1.20–1.40 m competition horses (8.27 ± 1.89 mmol/l) was significantly greater than the 1.10 m competition horses (6.40 ± 2.0 mmol/l) (*p* = 0.034). Horses that compete at 1.20 m–1.40 m are required to have a greater efficiency of movement and jump technique to facilitate a clean and quick round; in the present study horses competing at 1.20–1.40 m had a greater amount of experience (5 ± 2.18 years) competing at or above 1.10 m than horses competing at 1.10 m (3 ± 2.35 years). Over time, the greater workload over higher fences could change the proportion of aerobic to anaerobic muscle fibres, and thus could result in more work for the equine athlete even over smaller fences and basic flatwork. However, since show jumping involves finesse, fitness is not the only factor to consider when training these horses. A previous study [10] concluded that the quality of the jump produced by the horse would affect the workload of the horse and thus could affect the horse’s biochemistry; it has also been shown [6] that an increase in the height of the fences to be jumped had an effect on the biochemistry of the horses. Therefore, the objective method of assessment used in this report could help tailor exercise conditioning programs for trainers of show jumping horses.

The blood lactate concentrations showed an increase after the completion of the jumping phase, and markedly decreased after 60 min of rest (Table 1). Horses that competed at 1.40 m–1.50 m are reported to have a mean blood lactate value of 9.09 ± 0.09 l/l [2], which is much higher than the mean recorded during the standardized jumping test utilized in the present study. This difference in blood lactate could be the result of the difference in height of the obstacles studied (1.00–1.10 m vs 1.40–1.50 m) since the average velocities were similar in both studies; this infers that workload for the horse is proportional to fence height. The handheld analyser used in the present study has been shown to provide reliable results in horses and the data reported was within the linear range of the machine [29]. The other blood variables were measured using an analyser routinely used in equine medicine; to our knowledge, only its cardiac troponin assay has been validated in horses following the CLSI guidelines [30]. The venous blood pH, pCO2, and bicarbonate results should therefore be interpreted cautiously, keeping in mind that an increased PCV and variations in field conditions temperature can affect some analysers.

A limitation in the design of the present study is a potential fatigue effect by performing the standardized track exercise test 2 h after the completion of the standardized jumping course. Randomization of the exercise test order (performing either the jumping exercise *or* the standardized exercise test first) would have strengthened the study design However this was not possible due to logistical reasons including personnel availability, as well as access to the track and setting up the jumping course. Interestingly, it has been shown that even with passive recovery, horses with blood lactate levels of 2.7 mmol/L return to baseline (average 0.8 mmol/l) within minutes, as lactate clearance depends on primarily on cardiac output [31]. Therefore, it is unsurprising that heart rate and all blood variables assessed, including lactate, returned to baseline values – suggestive of sufficient recovery time - prior to the standardized track test. Similarly, the blood lactate values returned to baseline (0.1 ± 0.04 mmol/l) after the track warmup showing that the recovery was complete for the standardized track exercise.

The increase in PCV in equine athletes, including show jumpers, due to exercise is well documented [2]. In agreement with the literature, the PCV data collected showed an increase from baseline in PCV concentration at the conclusion of the jumping exercise, and a positive correlation with increasing velocities on the flat. Jumping induced an increase in PCV concentration that was not different from the one obtained after the track exercise at 5.31 ± 0.38 m/s, whilst the higher velocities induced a greater increase in PCV than the 1.10 m jumping course. The increase in PCV concentration associated with high intensity of jumping (44.4 l/l) and working at high speeds (5 m/s = 46.9 l/l, 8 m/s = 49.9 l/l and 11 m/s = 53.6 l/l) combined with the fact that the exercise was of short duration suggests that it was likely due to splenic contraction rather than dehydration [2]. We observed that the PCV increases progressively with speed in the track standardized exercise test (Table 1), which might be due to incomplete splenic contraction at lower speeds [32]. However, the maximum PCV reached in these horses is lower than previously reported (66 l/l) for Thoroughbred racehorses at maximal exercise [8]. This is probably due to a breed difference as well as a difference in the intensity of the exercise. Furthermore, the TP values only increased by a maximum of 10% at cessation of all exercise tests compared to baseline. A similar increase has been reported previously in Belgian Saddlebred show jumping horses over a 1.40 m course [33].

## Conclusions

Serial measurements of V200, V170, or VLa4 for the same horse can provide a safe and quantifiable measure of aerobic fitness which can be used for serial assessment of exercise conditioning under field conditions in Warmblood show jumpers. Data obtained during such tests can be compared “within” or “between” horses to assess fitness to compete, and performance. The data from the present study will help establish reference variables for show jumping horses tested using a standardized field test. This study also provides valuable information which enables the development of training programs that deliberately include flat work with a similar workload to a standardized jumping course, and therefore has the potential to decrease the risk of injury and optimize performance for this discipline.

## Methods

### Horses

Participants were recruited at show jumping competitions from the summer circuit in the Calgary, Alberta area. Although training schedules, competition schedules, and management differed, all horses were actively performing and were at a comparable stage of their individual show season. Regulations of the Canadian Equestrian Federation were followed by all participants. Owners or agents of the participants were asked to complete a written consent form and a standard questionnaire explaining the horse’s age, breed, diet, general exercise schedule, and show schedule for the jumping season, as well as the riders’ experience at 1.10 m, 1.20 m or higher, in addition to pertinent equine medical history. Horses were not reported to have a history of poor performance or lameness concerns.

Twenty-one healthy Warmblood horses with a mean age of 10.4 ± 2.6 years (range: 5–15 years) from ten different stables were studied. The study was performed in the summer during the show jumping season and all horses were actively competing between 1.10 m and 1.40 m categories. Overall, horses had 4 ± 2.3 years of experience jumping at or over 1.10 m. Of these, 10 horses competed at the 1.10 m level and 11 horses competed between 1.20 m and 1.40 m. The 1.10 m competition group had a mean age of 9.8 ± 2.9 years old and 3 ± 2.4 years of experience jumping at or over 1.10 m. The 1.20–1.40 m competition group had a mean age of 11.0 ± 2.4 years old and 5 ± 2.2 years of jumping experience at or over 1.10 m.

### Physical examination and equipment

Physical and lameness examinations were conducted on arrival at Bar None Ranches Inc., De Winton, Alberta. The facility comprised of both an indoor show jumping arena and an outdoor, five-furlong (1005.84 m) sand racetrack. The horse was left in a box stall for at least one hour to acclimatize. A portable cardiac ECG and GPS recording and transmitting device (500 Hz acquisition rate) (Televet100 system, Rösch; Samsung Galaxy S, Samsung) was then placed on the horse.

Each horse was equipped with 4 adhesive electrodes (Skintact FS-50, Leonhard Lang) positioned in a base-apex lead, and secured with an elastic band positioned around their girth. ECG monitors were attached to each horse, synchronized to a cell phone (Samsung Galaxy S, Samsung) on the rider’s wrist which provided GPS information and transmitted data to a computer. The ECG signal, heart rate and velocity data were displayed in real-time, and was recorded for analysis.

### Standardized jumping course and standardized incremental exercise test

Each horse completed both a standardized 1.10 m jumping course and one standardized exercise test after a resting period of at least 2 h in-between. The course was designed by a certified show jumping course designer and assembled in a 55 m × 55 m arena with a commercial footing (TravelRight Surfaces Inc). Horses were asked to follow a standardized warm up (3 min walk, 4 min trot, 2 min canter, 2 min riders’ choice on the flat, 4 show jumping fences). The horse and rider completed the standardized 360 m course with 12 obstacles set at 1.00 m–1.10 m high (10 verticals and 2 oxers). The horse was walked for 10 min to allow for recovery. After jumping, a minimum 2-h break was given to allow blood lactate concentrations to return to baseline before beginning the track standardized exercise test.

The racetrack was groomed on the morning of the standardized exercise test which was performed under dry conditions. The horses performed a standardized warm up on the track (3 min walk, 4 min trot and 1 min canter, 5 min walk). Horses were walked for 400 m and then worked for 600 m three times at 5 m/s, 8 m/s and 11 m/s; they were walked for 5 min between each trial. Riders were able to determine velocity accurately via the GPS unit attached to their arm. Except for two (out of 21) horses, the same rider (Weight: 72 kg) was used for both the jumping and track standardized exercise test portions of the trial. Each horse had its own English tack used for both exercise tests. Weather conditions were recorded.

### Sample collection

Blood samples were taken from the jugular vein, alternating sides. Seven jugular blood samples were collected for each horse. The first blood sample was taken directly after the first standardized warm-up, then directly after jumping and 60 min after jumping. Sample four was drawn after the second standardized warm-up. For the track standardized exercise test, blood samples were taken immediately after the 5 m/s and 8 m/s canters and 11 m/s gallops respectively. Rectal temperatures were recorded at the time of each blood sampling and temperature-corrected blood data is reported for pH, and pCO_2_ levels (Table 1).

A sodium heparin Vacutainer (BD) and an EDTA Vacutainer (BD) were filled at each sample. All sodium heparin Vacutainer (BD) samples were analysed trackside. A handheld system (iSTAT, Heska) using CG4+ cartridges (iSTAT, Heska) and corrected for rectal temperature provided blood pH, pCO_2_ and bicarbonate concentrations. Blood lactate concentrations were measured using another handheld analyser (Lactate Pro, Arkray). Samples were stored on ice. Packed cell volume (PCV, l/l) and total protein concentration (TP, g/L) were measured within twelve hours of collection.

### Data processing

Heart rate compared to velocity of the track standardized exercise test was used to calculate the slope of a linear-regression line and thus determine V200 and V170 (Fig. 1). Blood lactate compared to velocity from the track standardized exercise test was used to create an exponential non-linear regression curve to determine VLa4. Regression analyses were performed using Microsoft Excel 2013 (Microsoft).

### Statistical analysis

Statistical analysis was performed using a statistical program [34]. All data was reported as mean ± standard deviation (S.D.). For each measured physiological parameter (HR, blood lactate, pH, PCO2, bicarbonate), a mixed model ANOVA with Geisser-Greenhouse correction (therefore not assuming sphericity of the data) was used to study the differences between exercise tests (Standardized jumping, track standardized tests at 5 m/s, 8 m/s and 11 m/s) using the exercise test and the horse jumping level (1.10 m or 1.20–1.40 m) as fixed effects and the horses as random effect. A Dunnett post hoc test was used for multiple comparisons between the 3 track tests compared to the reference jumping test, and a Sidak post hoc test was used for multiple comparisons between the higher vs lower level horses in each exercise test. A QQ plot was used to confirm the assumption of normality, and the Geisser-Greenhouse’s epsilon value was used to confirm sphericity of the dataA non-parametric (Wilcoxon rank sum) test was used to compare the calculated physiological parameters (V200, V170, VLa4, curves slopes, which were all single measures) between the 1.10 m competition (*n* = 10) and 1.20–1.40 m competition (*n* = 11) groups at the same time point. Results were considered significant if *p* < 0.05.

## Data Availability

The datasets used during the current study are available from the corresponding author on reasonable request.

## Abbreviations

V170: The exercise velocity resulting in a heart rate of 170 beats per minute
V200: The exercise velocity resulting in a heart rate of 200 beats per minute
VLa4: The exercise velocity resulting in a blood lactate concentration of 4 mmol/L
PCV: Packed cell volume
TP: Total protein
HR: Heart rate
PCO_2_: Partial pressure of carbon dioxide
ANOVA: Analysis of variance
ECG: Electrocardiogram
